# Carotid Artery Stenosis and Ischemic Strokes in Patients with Giant Cell Arteritis: A Characteristic Pattern—Literature Review and Case Report

**DOI:** 10.1055/a-1704-0741

**Published:** 2022-01-28

**Authors:** Clemens Oerding, Frank Uhlmann, Johannes Wollmann, Ingmar Kaden, Kai Wohlfarth

**Affiliations:** 1 Department of Neurology, BG-Hospital Bergmannstrost, Halle (Saale), Germany; 2 Department of Radiology, BG-Hospital Bergmannstrost, Halle (Saale), Germany

**Keywords:** vasculopathy, stenosis, stroke, vessel wall enhancement, internal carotid artery, vasculitis, imaging, temporal artery biopsy, giant cell arteritis, Horton disease

## Abstract

**Purpose** Ischemic stroke is a relatively rare complication of giant cell arteritis often accompanied by vessel stenosis. Our purpose was to compare the location of internal carotid artery stenosis in GCA patients by performing a literature review suggesting a specific and characteristic pattern.

**Methods** We performed a PubMed research including all articles and cited articles reporting cases and case series about giant cell arteritis patients with internal carotid artery stenosis and ischemic strokes.

**Results** In this case series 39 cases were included. We found a clear tendency of giant cell arteritis-related stenosis to be in the intracranial segments (35/39 (89.7%)). Only in 8/39 (20.5%) patients there was further involvement of extracranial segments. Many cases (27/39 [69.2%]) showed a bilateral involvement.

**Discussion** This literature review reveals a specific pattern of internal carotid artery involvement in patients with giant cell arteritis and ischemic strokes. To our knowledge this pattern has not been reported as a sign strongly pointing toward giant cell arteritis before. We have not found case reports mentioning other common types of vasculitis reporting this involvement pattern.

**Conclusion** Internal carotid artery stenosis and ischemic stroke is a rare complication in patients with giant cell arteritis. Considering the characteristic features of bilateral distal internal carotid artery stenosis giant cell arteritis should be suspected which potentially leads to an early diagnosis and immunotherapy.

## Introduction

Ischemic stroke in giant cell arteritis (GCA) is a well-known complication. The incidence rate ranges between 2.8 and 7%. 1 2 3 4 5 6 7 Diagnosis can be challenging due to variable presentation and similarities with other inflammatory vessel diseases or causes of vascular stenosis.

In this literature review we want to discuss the characteristic localization of GCA-related bilateral intracranial internal carotid artery (ICA) stenosis comparing radiological and histopathological findings of previous studies. The possible challenges of diagnosing this disease will be demonstrated.

We want to present our own case of a 58-year-old female patient with suspected temporal-artery-biopsy-negative GCA who suffered recurrent bihemispheric strokes and hemodynamic impairment of both hemispheres while the only manifestation site was both intracranial carotid arteries. Despite immunosuppressive treatment the patient could not be prevented from experiencing new strokes.

## Literature Review

### Methods

Our aim was to perform a literature review of cases with internal carotid artery involvement in GCA and ischemic stroke. A PubMed research was performed including articles and cited articles about selected cases of patients describing stenosis of both or one ICA in patients with GCA and ischemic strokes at the time of diagnosis while searching for the terms “giant cell arteritis,” “GCA,” and “carotid artery,” “ischemic stroke” or “intracranial involvement.” The patients had to be diagnosed according to plausible clinical features and current guidelines, by a positive temporal artery biopsy (TAB) or histological proof of giant cells in other large vessels combined with clinical features attributable to GCA. Another criterion was that the segment or segments of stenosis were mentioned or could be figured out by analyzing the provided vascular imaging or autopsy.

Although other signs of inflammation can be drawn to attention 8 we have focused on stenosis, occlusion, or arterial wall thickening revealed by MRA, CTA, conventional angiography, or necropsy. Unless the original site of occlusion could be determined by sonography patients with bilateral proximal ICA occlusion or occlusion of the only involved ICA diagnosed by CTA, MRA or DSA were not patients since the original site of occlusion could be more distal. Moreover, cases with occlusions were considered if a necropsy was performed, and the maximum of inflammatory changes could be identified.

### Results

**Fig. 1:**
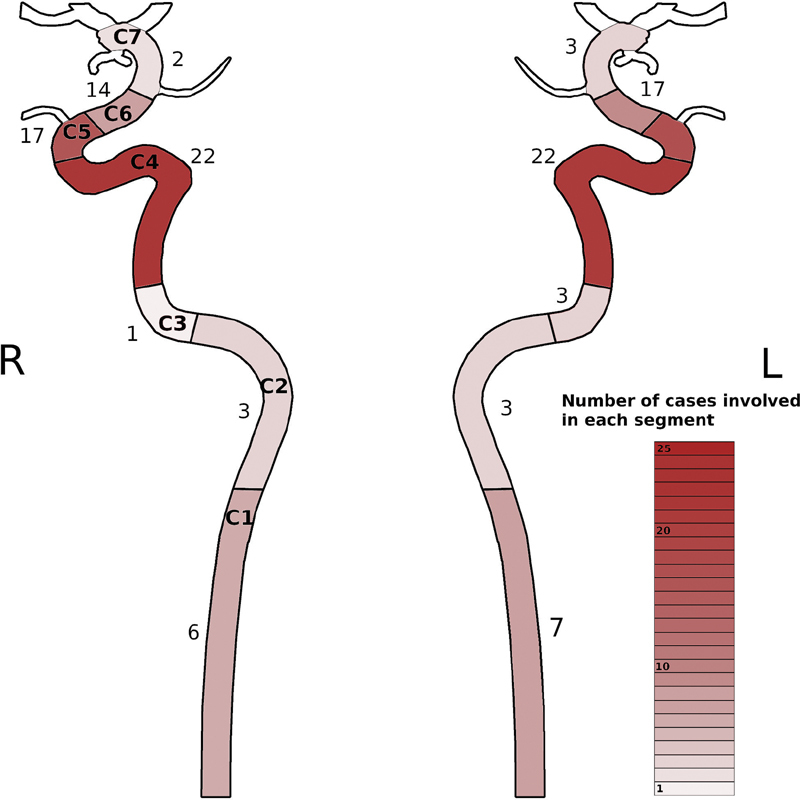
A schematic addition of all cases of ICA (internal carotid artery) involvement. A strong focus on the carotid siphons is apparent.

This literature review revealed a clear tendency of GCA to cause bilateral intracranial stenoses (mainly cavernous and [para]clinoid segment) in the case of ICA involvement. The patients' features are shown in Table 1 , a summary of the results in Table 2 . Bilateral and nearly symmetrical distal occurrence seems to be regular ( Fig. 1 ): in 27/39 (69.,2%) cases ICA involvement was bilateral. In four cases there was no information whether stenosis was bilateral. In 35/39 (89.7%) cases there was intracranial (all segments but C1 cervical segment) ICA stenosis. Only in 8/39 (20.5%) cases there was an involvement of extracranial segments. The rate of involvement in relation to the individual segments was C1 cervical segment 8/39 patients (20.5%), C2 petrous segment 5/39 patients (12.8%), C3 lacerum and C4 cavernous segment 27/39 patients (69.2%), C5 (para)clinoid segment 8/39 patients (20,5%), C6 ophthalmic (supraclinoid), and C7 communicating (terminal) segment 12/39 patients (30,8%). In contrast to previous findings, we could not confirm that the previously reported female dominance of the GCA large vessel variant 9 also accounts for ICA involvement (male/female = 22: 13; four cases not specified). A younger age compared with small vessel or only temporal artery GCA patients was apparent 9 : average 68.2 years, median 69 years.

**Table 1:** Case series of patients with GCA and stenosis of one or both ICA and ischemic strokes

Ref.	Neurological symptoms	Age [y] sex [m/f]	ESR [mm/h]	Angiography/Autopsy findings	Extracranial involvement	Intracranial involvement	Bilateral involvement	Diagnosis confirmed by
7	Headache, mild dysarthria, and a left beating horizontal nystagmus.	74 /m	61	Focal stenosis in the right carotid siphon (angiography)	No	Yes	No	Sonography, TAB
33	Headache, Horner syndrome, amaurosis fugax, headache	74 /m	n.a.	Moderate stenosis of the right cavernous and supraclinoid internal carotid artery (ICA; white arrow) and, to a lesser extent, the left ICA	No	Yes	Yes	TAB
	Headache, aphasia, right arm paresis	66 /m	n.a.	Both cavernous segment high-grade stenosis	No	Yes	Yes	TAB
	Headache, gait instability, amaurosis fugax, vision loss	79 /m	n.a.	High-grade stenosis of both cavernous/paraclinoid ICA	No	Yes	Yes	TAB
	Headache, wording difficulties, unsteadiness	59 /m	n.a.	Symmetric narrowing in both internal supraclinoid segments	No	Yes	Yes	TAB
	Right vision loss, hemiparesis	76 /f	n.a.	Stenosis of right carotid siphon	No	Yes	No	TAB
	Headache, dysarthria, imbalance	74 /m	n.a.	Right proximal and distal cavernous segment stenosis	No	Yes	No	TAB
22	Hemiplegia, neglect, headache	59 /m	59	Narrowing of both intradural ICA ending at the intracranial bifurcation (angiography)	No	Yes	Yes	TAB
34	Global aphasia	65 /m	110	Bilateral supraclinoid portions	No	Yes	Yes	TAB
35	n.a.	72 /f	98	Bilateral carotid siphon stenosis (angiography)	No	Yes	Yes	Sonography, TAB
36	Self-limited upper limb weakness, facial droop, no headache	59 /f	97	Stenosis in the ophthalmic segment of the left ICA (angiography)	No	Yes	No	TAB
	Vision loss, quadrantanopia, headache	72 /f	50	Bilateral carotid siphon stenosis (angiography)	No	Yes	Yes	TAB
	Vertigo, nausea, sweating, headache	69 /m	21	Multiple stenoses in the intracranial left ICA (angiography)	No	Yes	No	ACR criteria
	Gait instability, poor limb coordination, headache	73 /m	68	Narrowing of the cavernous segments of both internal carotid arteries (angiography)	No	Yes	Yes	Sonography, clinical symptoms
6	n.a.	n.a.	n.a	Extracranial stenosis >60% (uni- or bilateral not mentioned)	Yes	No	No	n.a
	n.a				Yes	No	No	n.a
	n.a				Yes	No	No	n.a
	n.a				Yes	No	No	n.a.
37	Headache, hemiparesis, aphasia, apraxia	75 /m	74	Left supraclinoid segment stenosis	No	Yes	No	TAB
	Headache, hemiparesis, dysarthria	70 /f	108	Bilateral supraclinoid and petrous segment stenosis	No	Yes	Yes	Not mentioned
25	Vision loss, headache	66 /m	14	Bilateral stenosis of petrous and cavernous segments (angiography)	No	Yes	Yes	TAB
24	Frontal lobe syndrome, gait ataxia, headache	61 /f	unknown	Circumferential arterial wall thickening of carotid siphons (angiography)	No	Yes	Yes	TAB
38	Blindness, hemiparesis, ataxia, headache	67 /f	99	Bilateral intracranial stenosis of cavernous and paraclinoid segments	No	Yes	Yes	TAB
39	Episodic double vision and visual blurriness, headache	59 /m	50	Bilateral stenosis of the carotid siphons (angiography)	No	Yes	Yes	TAB
40	Transient aphasia, headache	69 / m	106	Left-sided stenosis of the cervical segment and multifocal stenosis of the carotid siphon and cavernous segment (angiography)	Yes	Yes	No	TAB
41	Transient palsy and dysphasia, scalp tenderness, no headache	69 /f	86	Bilateral stenosis of the carotid siphons (angiography)	No	Yes	Yes	TAB
42	Progressive cognitive decline, drowsiness, headache	75 /f	unknown	Obstruction of both internal carotid arteries at the siphon (angiography)	No	Yes	Yes	TAB
43	Hemiparesis, tenderness of head, neck and scrotum, headache	61 /m	129	Bilateral stenosis of the carotid siphons (angiography)	No	Yes	Yes	Giant cells in biopsy of neck and occipital arteries
44	Ischemic optic neuropathy, headache	60 /f	64	Bilateral carotid siphon arteritis (angiography)	No	Yes	Yes	TAB
12	Diplopia, gait disturbance, Horner's syndrome, hemiparesis, headache,	60 /m	43	Bilateral ICA-stenosis of the full length with maximum in siphons, signs of inflammation, and giant cells found in both ICA (autopsy)	Yes	Yes	Yes	Giant cells in ICA (autopsy)
1	Brachiofacial palsy, no headache	65 /f	67	Proximal bilateral occlusion (angiography), proliferation of initima, and giant cells in both cavernous segments—(autopsy)	No	Yes	Yes	Giant cells in ICA (autopsy)
23	Palsy and ataxia, headache	74 /m	60	Mild involvement of both carotid sinuses (autopsy)	No	Yes	Yes	TAB
	Blindness, dysphasia, hemiparesis, headache	80 /m	80	Left siphon occlusion, left cervical part inflammation, and right siphon inflammation without stenosis (autopsy)	Yes	Yes	Yes	TAB
	Vertigo, blindness, headache	79 /m	58	Mild bilateral siphon inflammation, right-sided cervical course mild inflammation (autopsy)	Yes	Yes	Yes	TAB
	Lateral medullary syndrome, headache	75 / m	47	Stenosis of both cavernous segments (autopsy)	No	Yes	Yes	TAB
45	Ischemic optic neuropathy, hemiparesis, headache	61 /f	45	Long stenotic area in the intracranial part of the left ICA (angiography); GCA in both STA, ICA, ECA, and basilar artery (autopsy)	No	Yes	No	TAB
46	Blindness, headache	68 /m	119	Giant cells and reduction of lumen on both sides at the origin of the ophthalmic arteries (autopsy)	No	Yes	Yes	TAB
47	Hemiplegia, headache	59 /f	Unknown	Stenoses of both upper ends of intraosseous parts ending at bifurcation with lymphocytes and giant cell infiltration (autopsy)	No	Yes	Yes	Giant cells in ICA (autopsy)
	Hemianopia, ocular motor disturbance, hemiparesis, headache	63 /m	Unknown	Both intracranial parts in neighborhood of anterior clinoid processes with lymphocytes and giant cell infiltration (autopsy)	No	Yes	Yes	Giant cells in ICA (autopsy)

Abbreviations: EMS, encephalo-myo-synangiosis; ICA, internal carotid artery; MCA, middle cerebral artery; STA, superficial temporal artery; TAB, temporal artery biopsy.

**Table 2:** 

Cases	New onset headache [%]	Extracranial ICA stenosis [%]	Intracranial ICA stenosis [%]	Bilateral ICA stenosis [%]	Ratio male/female	Patient age	
						Median [years]	Mean [years]
39	32/34 [94] (five patients with no information about symptoms)	8/39 [20]	35/39 [90]	27/39 [69]	22:13 (four cases not specified)	69	68.2

### Case Report

**Fig. 2:**
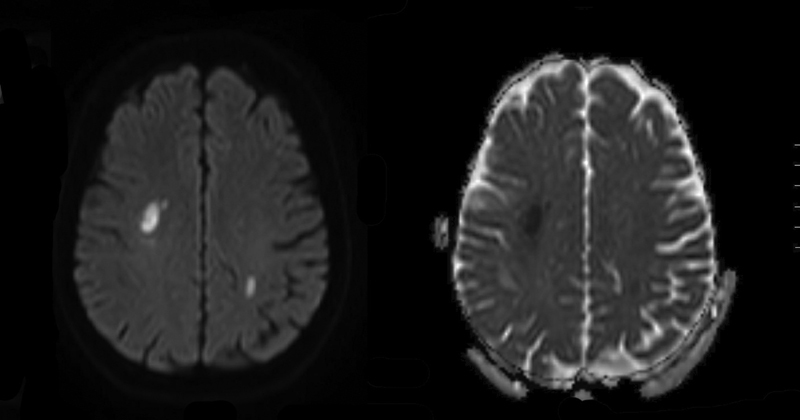
MRI on the left side diffusion-weighted imaging (DWI), on the right side apparent diffusion coefficient (ADC) showing bilateral infarcts at the time of the initial hospital admission.

**Fig. 3:**
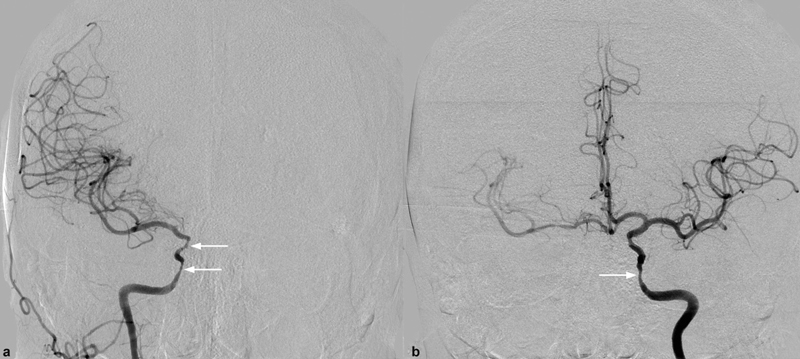
Conventional angiography of the ( **a** ) right and ( **b** ) left ICA showing smooth stenosis of both C3-C6 segments and a partial supply of the right middle and anterior cerebral artery territories by crossflow at the anterior communicating artery. ICA, internal carotid artery.

**Fig. 4:**
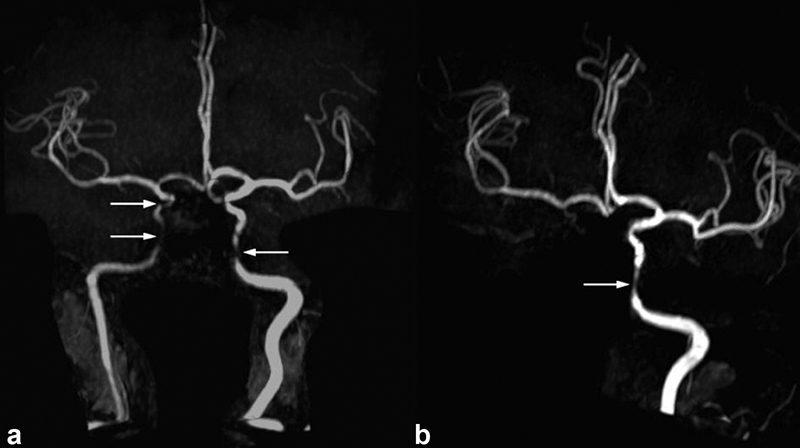
MR-angiography (time of flight imaging) ( **a** ) of the first hospital admission showing bilateral ICA stenosis in the C3–C6 segments, ( **b** ) performed 4 months later revealing right-sided ICA occlusion and left-sided stenosis consistent with ( **a** ). ICA, internal carotid artery.

**Fig. 5:**
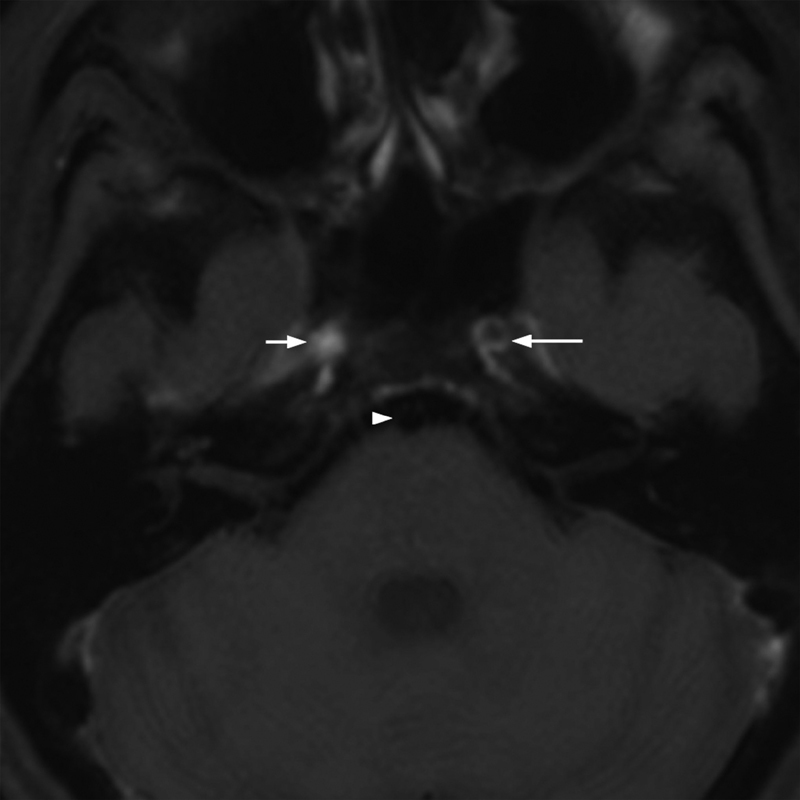
T1 black-blood post-gadolinium imaging sequence MRI showing normal basilar artery ( *arrowhead* ), left ICA in the cavernous segment with vessel wall enhancement (VWE, *long arrow* ) and occluded right ICA with hyperintense thrombus ( *short arrow* ). ICA, internal carotid artery.

**Fig. 6:**
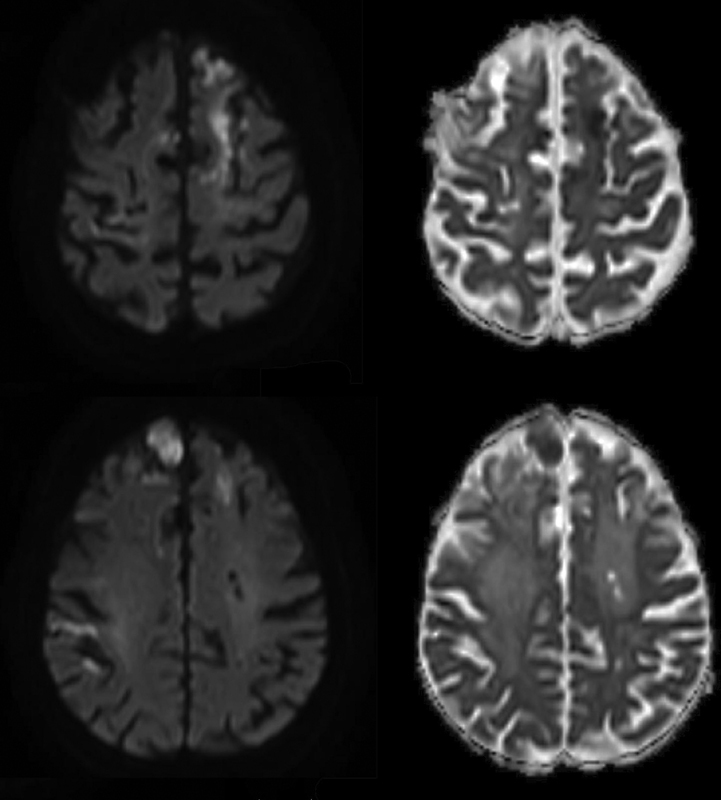
MRI on the left side diffusion-weighted imaging (DWI), on the right side apparent diffusion coefficient (ADC) showing multiple bilateral infarcts of various age as a result of progressive disease in the course of 1 year and 8 months after the first hospital admission.

A 58-year-old female patient was admitted to the emergency room complaining of sudden onset palsy of the left arm and leg. No headache was present. She had a history of hypertension. We diagnosed a diabetes mellitus type II and dyslipidemia. CT angiography and MRI/MR angiography revealed bilateral stenosis of both intracranial ICA pronounced on the right side, a perfusion deficit of the right hemisphere and bilateral new infarcts also pronounced on the right side. A diagnostic cerebral angiography, and MRI showed bilateral infarcts and smooth and mostly concentric bilateral distal ICA stenosis, a 70% stenosis of the right internal carotid artery in the C3 to C6 segments, a 60% stenosis of the left ICA in the same segments, and a partial supply of the right middle and anterior cerebral artery territories by crossflow at the anterior communicating artery ( Figs. 2 , 3 and 4a ). Laboratory investigation revealed an elevated blood sedimentation rate (99 mm/h), C-reactive protein (79 mg/L), and ANA titer (1:640). Antibodies found in rheumatic diseases repeatedly tested negative. CSF was normal. Testing for HIV, VZV, and hepatitis B/C was unremarkable. We discussed primary angiitis of the central nervous system (PACNS) as differential diagnosis but since CSF was normal and BSR and CRP elevated, there was no further involvement of vessels apparent in the conventional angiography other than both ICAs; a brain biopsy was unrevealing and complete vessel occlusions tend to be rare in PACNS. 10 GCA seemed more likely. No other cause for the ischemic strokes, such as atrial fibrillation, could be found. Four months later new infarcts on the right side and an occlusion of the right ICA were found (Fig. 4b). Duplex sonography of cranial arteries according to EULAR recommendations 11 and a whole body PET-CT in search of large vessel involvement (e.g., aortitis) were unrevealing; however, the latter was performed during steroid treatment. A TAB on the right side was performed showing no results of inflammation. A biopsy of the stenotic or occluded carotid artery region was not feasible. Facing progressive ischemic strokes and vessel stenoses we decided to start a steroid treatment suspecting GCA under which BSR and CRP decreased. An MRI (T1 black-blood post-gadolinium imaging sequence, Fig. 5 ) revealed left-sided ICA vessel wall enhancement (VWE) of the cavernous and petrosal segments which gave us a cause to suspect focal arterial inflammation. After treatment with methotrexate, prednisolone and later tocilizumab inflammatory parameters were lowered. However, despite immunotherapy new infarcts occurred ( Fig. 6 ). We decided to present this case although the patient could not be diagnosed with GCA by TAB and the diagnosis is not certain but due to clear signs of inflammation in both distal ICAs there are significant similarities with our case reviews' characteristic pattern of involvement.

## Discussion

We found that internal carotid artery involvement in GCA with ischemic strokes follows a characteristic pattern with bilateral mostly symmetrical distal ICA stenosis or occlusion (typically cavernous and clinoid segments).

To our knowledge this is the first systematic review examining case series and case reports about GCA patients with ischemic stroke and ICA stenosis/occlusion. This bilateral distal internal carotid artery involvement pattern was mentioned before to be a possible manifestation in GCA patients 12 but not yet suggested as a strong diagnostic hint toward GCA which should lead to further investigation in acute stroke patients, e.g., TAB, sonography, PET-CT, or MR angiography to support clinical suspicion. 13 14

It has been reported before that patients with large-vessel-giant-cell-arteritis have less headache, jaw claudication or visual symptoms, are younger than GCA patients with temporal arteritis and their TAB specimens are less likely to yield positive results 8 all of which are essential findings and symptoms for the diagnosis according to current GCA guidelines. To date there is no diagnostic proof but positive biopsy and proof of giant cells which in the case of only ICA involvement is often not feasible. Diagnosing GCA following ACR criteria 48 used to be common years ago but is considered obsolete nowadays. According to the current German guidelines on the management of GCA 15 diagnosis should be made by an experienced interdisciplinary team that considers laboratory and radiological findings as well as suggestive clinical features besides histological proof in its diagnostic work-up. Moreover, we want to emphasize the pivotal role of cranial artery sonography as well as positron emission tomography (PET) in search of large vessel involvement several of the cases we included referred explicitly to the obsolete 1990 ACR criteria to confirm diagnosis. Furthermore, the understanding of symptoms and inflammatory distribution of GCA changed in the course of time so that previous cases might have been misdiagnosed with a higher probability or confused with other types of vasculitis that were less well researched at that time such as PACNS. That accounts for clinical diagnostic precision and for the interpretation of histological specimens especially since giant cells are not a phenomenon exclusively observed in GCA but also (for e.g.,) in PACNS patients. 16 Sensitivity might be compromised by the existence of unusual phenotypes. As a confounding factor cases of proximal ICA stenosis might be solely and coincidingly due to macroangiopathy without giant cell infiltration such as presumably in the cases of de Boysson et al. 6 Plenty of data about vessel involvement in CGA is available but mostly in case reports and smaller series the exact localization of stenosis within the carotid artery is not clarified which lowered the number of included articles. Other patients with ICA stenosis were not included since no ischemic stroke was detected, for e.g., 17 four cases that showed an ICA involvement at the carotid siphons.

We scarcely found comparable report cases in other categories of vasculitis (e.g., TAK, polyarteritis nodosa, Kawasaki disease, ANCA-associated small vessel vasculitis, PACNS, sarcoidosis, Behcet's disease, and varicella zoster virus vasculopathy). In one case of Behçet's disease a patient had bilateral proximal ICA occlusion. 18 Patients with TAK which is increasingly considered as a spectrum disease along with GCA 19 did not show any internal carotid artery involvement without continuous affection of the common carotid artery in all cases of a recent study. 20

To a lesser degree ICA stenosis can occur in the short proximal intradural course but rarely involves purely intradural vessels. 17 21 22 This “intra-/extradural border” might be caused by different arterial wall features of the intra- and extracranial arteritis. Intradural arteries tend to have much thinner vessel walls with less elastin. Wilkinson and Russell suggested this difference to be the reason of the intradural sparing of GCA since vessel wall elastin is considered to be a major target of inflammation in GCA, 21 23 however, vessel wall elastin may extend up to 5 mm intradurally 6 which might explain a variable involvement of intradural internal carotid arteries. 21 Several cases reporting intradural wall thickening or stenoses of vertebral arteries in GCA patients can be found. 23 24 25 It is noteworthy that using MRI imaging diagnosis of vessel wall inflammation itself can be challenging. According to a recent work of Guggenberger et al 26 VWE caused by prominent vasa vasorum might be confused with large artery inflammation of the proximal intradural ICA and vertebral arteries in elderly subjects.

The incidence rate of ischemic stroke in patients with GCA has been repeatedly reported ranging between 2.8 and 7%. 1 2 3 4 5 6 7 The precision of determining this incidence rate might be influenced by the fact that also a TAB has a sensitivity between 70 and 90%. Consequently, the incidence rate of GCA-related strokes might have been underestimated in previous studies. Also, the typical features of GCA such as temporal headache and jaw claudication might be caused by different inflamed arteries than those causing ischemic strokes which might add to the underestimation of incidence rate. Interestingly, Cid et al 27 found that a hemoglobin level as a marker of chronic inflammatory response is associated with a lower risk of cerebral ischemic complications. The authors assumed that an intensified neovascularization could be the consequence of inflammation and protective against neural damage in the case of ischemic stroke. Gonzalez-Gay 28 investigated this further and found lower circulating vascular endothelial growth factor in vivo and lower VEGD transcription in patients with severe occlusive disease. Hočevar et al 29 discovered that a higher CRP value increases the risk of ischemic stroke in GCA patients with a similar explanation as the aforementioned authors: “through a local angiogenic function of proinflammatory cytokines.

It has been repeatedly reported that at the time of diagnosis patients are more likely to have an ischemic stroke in the vertebrobasilar region rather than carotid perfused region with an estimated ratio of around 5:1 2 6 28 30 which changes to a significant predominance of ischemic strokes in the anterior circulation months or years after diagnosis. 28 This might represent an approximation of the vertebral/carotid artery stroke ratio to the normal population. Compared with ICA the vertebral artery pattern of involvement seems to be less characteristic and less predictable. 23 According to these findings the typical GCA patients with ischemic stroke were described as “old men” with cardiovascular risk factors and strokes in the vertebrobasilar territory. 2

Stenosis and VWE might pose a higher risk for ischemic strokes in GCA patients but has not been studied systematically yet. Caselli and Hunder 1988 investigated the occurrence of ischemic strokes in GCA patients during a 3-year study period and found a higher incidence rate of ischemic strokes in patients with carotid disease; however, the latter was defined only by bruits and/or diminished pulses. To our knowledge there are no studies comparing the degree of stenosis (including VWE) with the incidence rate of ischemia. Early treatment seems to be important: a retrospective database study showed a strong focus of GCA-related strokes with a fivefold-increased risk during the active phase of the disease. 3 These findings suggest the necessity of an immediate and effective treatment after diagnosis. Diabetes and hypertension which are known to be independent risk factors for cardiovascular ischemia seem to add to the risk of ischemic strokes during the follow-up of 6 months after diagnosis of GCA. 31

## Conclusion

As mentioned by the Chapel Hill Consensus Conference 2012 authors, “if the features of a vasculitis that is confined to one organ indicate that it is a limited expression of one of the systemic vasculitides, this vasculitis should be considered a limited expression of that category of vasculitis rather than an independent SOV (single organ vasculitis).” 32 We want to emphasize that the knowledge of this characteristic involvement pattern of GCA could help to find the right diagnosis in similar patients. This could lead to an earlier immunotherapy and a better outcome of the respective patients.
